# Harmonizing AI governance regulations and neuroinformatics: perspectives on privacy and data sharing

**DOI:** 10.3389/fninf.2024.1472653

**Published:** 2024-12-17

**Authors:** Roba Alsaigh, Rashid Mehmood, Iyad Katib, Xiaohui Liang, Abdullah Alshanqiti, Juan M. Corchado, Simon See

**Affiliations:** ^1^Department of Computer Science, Faculty of Computing and Information Technology (FCIT), King Abdulaziz University, Jeddah, Saudi Arabia; ^2^Faculty of Computer and Information Systems, Islamic University of Madinah, Madinah, Saudi Arabia; ^3^Department of Computer Science, University of Massachusetts, Boston, MA, United States; ^4^BISITE Research Group, University of Salamanca, Salamanca, Spain; ^5^Air Institute, IoT Digital Innovation Hub, Salamanca, Spain; ^6^Department of Electronics, Information and Communication, Faculty of Engineering, Osaka Institute of Technology, Osaka, Japan; ^7^NVIDIA AI Technology Center, NVIDIA Corporation, Santa Clara, CA, United States

**Keywords:** neuroinformatics, privacy, data sharing, ethical AI, AI governance, regulatory frameworks, data standardization, interoperability

## 1 Introduction

In the rapidly evolving field of neuroinformatics, the intersection of artificial intelligence (AI) and neuroscience presents both unprecedented opportunities and formidable ethical challenges (Ienca and Ignatiadis, 2020; Dubois et al., 2023; Parellada et al., 2023; Scheinost et al., 2023). As AI technologies increasingly underpin neuroscientific research, it is crucial to establish robust governance frameworks that not only match the ambitious scope of this research but also adhere to stringent requirements for privacy and data sharing (Eke et al., 2022; Jwa and Martinez-Martin, 2024; Yuste, 2023; UK Government, 2018). This paper explores the urgent need to harmonize AI governance regulations with neuroinformatics practices, with a specific focus on the domains of data sharing and privacy.

This opinion article is grounded in a comprehensive analysis of over 4,000 research articles and AI regulation documents, supplemented by referencing over 100 pivotal articles and documents. It offers a critical examination of current AI governance frameworks and the existing challenges at the intersection of AI and neuroinformatics.^1^ Through this analysis, we systematically explore the state-of-the-art in neuroinformatics (Section 2), its challenges (Section 3), and the evaluation of AI governance (Section 4), identifying key alignments and gaps (Section 5). We conclude with strategic recommendations for better integration of these fields, aimed at enhancing research outcomes while ensuring privacy and fostering ethical practices (Section 6).

By integrating these diverse perspectives, the paper aims to spark a constructive dialogue among policymakers, researchers, and practitioners. The objective is to develop a cohesive framework that not only supports innovation in neuroinformatics but also operates under the umbrella of conscientious and effective AI governance, ensuring that neuroinformatics can continue its rapid advancement in a responsible and ethically sound manner.

## 2 State-of-the-art in neuroinformatics

Neuroinformatics has experienced transformative advancements through enhanced data sharing frameworks and technological innovations (Daidone et al., 2024; Weiner et al.2015; MacGillivray et al., 2018; Cao et al., 2023). These developments have significantly improved research efficiency and fostered innovation, particularly in complex areas such as autism (Parellada et al., 2023; Zucchini et al., 2023; Saponaro et al., 2022) and Alzheimer's disease (Yao et al., 2023; Zhang et al., 2022; Dubois et al., 2023).

One of the most notable advancements in neuroinformatics is the standardization of data sharing practices (Wang J. et al., 2023; Alzheimer Europe, 2021). Initiatives such as the Alzheimer's Disease Neuroimaging Initiative (ADNI) (Weiner et al., 2015a,b) and the Common Data Element (CDE). Project in epilepsy research (Loring et al., 2011) exemplify how standardized practices, including shared ontologies, common data elements, and standardized data formats, facilitate robust validation of results across diverse studies and enable large-scale, multi-center studies (Wang L. et al., 2023; MacGillivray et al., 2018; Yaseen et al., 2023). These elements are fundamental for integrating data from various sources, evident in the success of these projects (Ojo et al., 2020; Viejo et al., 2023). This integration is vital for the scalability and reproducibility of neuroinformatics research, leading to more reliable outcomes and faster scientific progress (Gurari et al., 2015; Baker et al., 2015; Sarwate et al., 2014).

Technological enhancements such as electronic health records and sophisticated data repositories have revolutionized how data is collected, managed, and shared within the field (Gentili et al., 2021; Leoratto et al., 2023). These technologies are crucial for supporting longitudinal studies and comprehensive data analyses necessary for understanding long-term outcomes of neurological conditions including traumatic brain injury (Vallmuur et al., 2023; Yaseen et al., 2023). Moreover, the role of international collaborations cannot be overstated. Initiatives such as the Dominantly Inherited Alzheimer Network (DIAN) (Bateman et al., 2012) and global epilepsy research consortia (Galanopoulou et al., 2021; Mishra et al., 2022) highlight the importance of pooling resources and expertise to tackle complex scientific questions, significantly enhancing the scope and impact of research efforts (Chou et al., 2022). Privacy-preserving technologies including differential privacy, encryption, anonymization, and blockchain have become integral to maintaining data confidentiality, while enabling expansive research and clinical applications (Zhang Z. et al., 2023; Yuste, 2023; Yang et al., 2023; Patel et al., 2023). Notably, federated learning and edge computing have gained attention for their role in supporting decentralized research models while ensuring privacy (Zou et al., 2023; Yang et al., 2024; Mitrovska et al., 2024). These technologies enable researchers to collaborate without compromising the security of sensitive data, crucial in neuroinformatics where privacy concerns are paramount (Gong et al., 2022; Selfridge et al., 2023; Cali et al., 2023).

## 3 Challenges in neuroinformatics

The landscape of neuroinformatics is fraught with complex challenges that stem from the integration of advanced data sharing, privacy, and security considerations (White et al., 2022; Sarwate et al., 2014). These challenges are crucial to address as they directly impact the efficacy and ethical alignment of neuroinformatics research (Ienca and Ignatiadis, 2020).

Resistance to data sharing remains a primary obstacle, often fuelled by concerns over data ownership and the potential for misuse (Tudosiu et al., 2022). This resistance necessitates clear policies that balance intellectual property rights with the need for open access to data (Redolfi et al., 2023). Additionally, the traditional academic reward system, which prioritizes individual achievements over collaborative efforts, further discourages open data sharing (Versalovic et al., 2023). Technical challenges such as managing and standardizing large, complex datasets add another layer of difficulty. Data heterogeneity, varying formats, and the necessity for robust metadata standards complicate data integration and utilization across various research platforms, making it challenging to achieve consistent and reliable research outcomes (Wang L. et al., 2023; Yang et al., 2024).

Privacy and security in neuroinformatics, particularly in neuroimaging, face unique challenges due to the technical complexity and resource demands of deploying privacy-preserving technologies such as federated learning and advanced encryption methods at scale (Xie et al., 2023; Zhu et al., 2023; Yu et al., 2023; Ay et al., 2024; Zhang C. et al., 2023). Balancing privacy with data utility is critical, as techniques including anonymization must not compromise the usefulness of data for medical research and diagnosis (Patel et al., 2023; Cali et al., 2023). Continuously developing robust security measures is essential to protect data from adversarial attacks and unauthorized access (Zhao et al., 2024).

Advancing neuroinformatics also requires substantial resources and infrastructure, including secure data repositories, high-performance computing facilities, and efficient data-sharing platforms, which support large-scale initiatives and sophisticated data analysis (Zhu et al., 2023; Yu et al., 2023; Viejo et al., 2023). These resources enable not only cutting-edge research but also the implementation of technologies including blockchain and federated learning, which demand considerable computational power (Xia et al., 2023; Tozzi et al., 2023; Ay et al., 2024; Yang et al., 2023). The significant investment and logistical challenges associated with these technologies often limit their widespread adoption, impacting the field's ability to ensure data privacy and manage large datasets effectively (Li et al., 2020).

## 4 AI governance regulations

AI governance guidelines across regions such as the European Union (EU), United States (USA), United Kingdom (UK), and China, along with global organizations, showcase diverse approaches to privacy preservation, data sharing, and ethical management of AI technologies (European Commission, 2021; POTUS, 2023; Standing Committee of the National People's Congress, 2016; Metcalfe et al., 2024; European Parliament, 2024).

The EU's AI Act regulates AI systems based on risk levels and emphasizes transparency, accountability, and stakeholder engagement to foster a human-centric AI ecosystem. It categorizes AI systems into various risk levels, with specific obligations designed to safeguard rights, health, safety, and promote innovation (European Parliament, 2024; European Union, 2024). The USA employs various frameworks and acts (The White House, 2023, 2022; National Telecommunications and Information Administration, 2023; National Security Commission on Artificial Intelligence, 2021), such as the Executive Order on Safe and Trustworthy AI (POTUS, 2023), which focuses on AI standards, research, and ethical deployment. The AI Risk Management Framework by NIST outlines strategies to manage AI risks, emphasizing resilience, fairness, and transparency (NIST, 2023).

The UK's AI framework balances innovation with protection, governed by the AI Authority which ensures compliance with safety, transparency, fairness, and governance standards (Tobin, 2024; UK Government, 2024). This framework supports AI assessments and promotes international regulatory interoperability (House of Lords Select Committee on Artificial Intelligence, 2018; AI Safety Institute, 2024; Metcalfe et al., 2024). China emphasizes lawful data collection and stringent security measures within its AI regulations, presenting unique challenges for cross-border data transfers (The National New Generation Artificial Intelligence Governance Specialist Committee, 2021; The State Council of the People's Republic of China, 2017; Webster et al., 2017). These regulations are part of a broader strategy to balance technological innovation with ethical governance (China Briefing Team, 2021; Standing Committee of the National People's Congress, 2016; Roberts et al., 2021; Wu et al., 2020; Sheenhan, 2024).

While the EU, UK, and USA share a focus on promoting ethical standards and transparency (European Commission, 2024), the EU's comprehensive regulatory framework contrasts with the more decentralized, state-based approaches seen in the USA. The UK's strategy intermediates these approaches with a centralized authority that still encourages innovation (Tobin, 2024). China's approach emphasizes stringent security and data localization (Standing Committee of the National People's Congress, 2016), representing a distinct paradigm that requires careful navigation to align with Western data privacy norms and open AI research methodologies (Roberts et al., 2021). Organizations such as OECD (2024b,a) and UNESCO (2023) set global standards for ethical AI practices, advocating for human rights, transparency, and international cooperation, which aim to bridge regional differences and foster a unified approach to AI governance.

## 5 AI governance regulations and neuroinformatics: alignment, gaps, and challenges

The integration of neuroinformatics within global AI governance frameworks reveals a robust alignment, especially in privacy and data protection (Wang J. et al., 2023; Tozzi et al., 2023). Initiatives such as the ADNI (Weiner et al., 2015a,b) and the CDE Project in epilepsy research (Loring et al., 2011) demonstrate compliance with international privacy regulations such as the GDPR (European Union, 2016; Alzheimer Europe, 2021; White et al., 2022; Muchagata et al., 2020). These efforts underscore a commitment to safeguarding sensitive health data and adhering to high ethical standards (Alzheimer Europe, 2021). Ethical considerations in neuroinformatics strongly resonate with the principles outlined in frameworks such as the EU's AI Act (Stahl and Leach, 2023). Neuroinformatics practices, particularly in handling data related to genetic research and brain-computer interfaces (BCIs), strive to align with these governance frameworks, ensuring informed consent (Bannier et al., 2021) and cognitive liberty (Schiliro et al., 2023) as central to their operations (Kulynych, 2002; Ligthart and Meynen, 2023; Hemptinne and Posthuma, 2023).

Despite these alignments, significant gaps persist, particularly in data standardization and interoperability (Daidone et al., 2024; Wang J. et al., 2023). The lack of unified data formats and protocols across international borders complicates efforts in global neuroinformatics collaborations (Zuk et al., 2020; Mulugeta et al., 2018). For instance, the variability in data management practices hinders the ability to maintain consistent transparency and accountability, making it challenging to comply fully with AI governance regulations across jurisdictions (Cheung et al., 2023; Yi et al., 2020). Additionally, data localization laws in countries, including China (Ministry of Science and Technology China, 2021; The National New Generation Artificial Intelligence Governance Specialist Committee, 2021; The State Council of the People's Republic of China, 2017; Webster et al., 2017), introduce complexities that may affect the unrestricted exchange of neuroinformatics data and adherence to international standards (Liu et al., 2022; Acar et al., 2023; Chou et al., 2022). These regulations highlight the need for careful navigation to facilitate global research collaborations, which are essential for advancing the field (Ownbey and Pekari, 2022; Russell et al., 2023).

Technologies including federated learning (Zhao et al., 2022; Sun and Wu, 2023) and blockchain (Song et al., 2023; Singh and Jagatheeswari, 2023; Yang et al., 2023) are emphasized in AI governance for enhancing data security (Kharat et al., 2014; Higuchi, 2013). However, neuroinformatics often struggles with the practical implementation of these technologies due to inconsistent regulatory support and the nascent state of these technologies in practical, research-focused environments (Zhu et al., 2023; Yu et al., 2023). The need for interdisciplinary collaboration is highlighted by the complex ethical, legal, and technical challenges in neuroinformatics (Farah, 2005; Blinowska and Durka, 2005; Wajnerman Paz, 2022). Current AI governance frameworks sometimes lack the flexibility to accommodate the rapid pace of technological advancements in neuroinformatics, necessitating ongoing revisions to ensure they remain relevant and effective (Jwa and Martinez-Martin, 2024; Yuste, 2023).

## 6 Discussion: harmonizing AI governance and neuroinformatics

Technological advancements such as federated learning, edge computing, and advanced anonymization techniques have shown substantial potential to align with stringent privacy regulations and foster ethical AI usage in neuroinformatics (Wang and Gooi, 2024; Zhang Z. et al., 2023; Zhu et al., 2023; Yu et al., 2023). Despite their promise, the application of these technologies has been uneven, highlighting a gap between technological capability and its practical implementation. Investing in dynamic consent mechanisms and robust data governance practices is crucial (Eke et al., 2022). These innovations are indispensable for progressing neuroimaging research without compromising privacy or ethical standards, ensuring that technology implementation keeps pace with regulatory expectations and community trust (Jwa and Martinez-Martin, 2024; Yuste, 2023).

The preservation of cognitive privacy (Schiliro et al., 2023) and the management of informed consent are pivotal in neuroinformatics, requiring ongoing attention to align with evolving ethical standards (Kulynych, 2002; Ligthart and Meynen, 2023; Hemptinne and Posthuma, 2023). These considerations are crucial as they govern how sensitive data, especially neural data, is handled. Enhancing public awareness and promoting interdisciplinary research are vital for ensuring that stakeholders are well-informed and that technologies interacting with sensitive data are developed responsibly (Green, 2015). This approach supports a transparent dialogue between researchers and the public, fostering trust and facilitating ethical advancements in neuroinformatics (Wardlaw et al., 2011; Illes and Reiner, 2015).

Regulatory complexities, especially those arising from national security concerns and data localization laws, significantly impact international collaboration in neuroinformatics (Ownbey and Pekari, 2022; Russell et al., 2023). These laws can stifle the global exchange of data and insights, critical for advancing the field. Developing unified standards that cater to diverse regulatory environments, such as those in the USA (POTUS, 2023; The White House, 2023, 2022; National Telecommunications and Information Administration, 2023; National Security Commission on Artificial Intelligence, 2021; NIST, 2023) and the EU (AI and Partners, 2024; Council of Europe - Commissioner for Human Rights, 2019; European Commission, 2021; European Parliament, 2024), is essential. Such standards would not only streamline compliance processes but also enhance global research initiatives (Ownbey and Pekari, 2022; Russell et al., 2023) by promoting data interoperability across jurisdictions. Addressing these regulatory challenges is fundamental to fostering a collaborative international research environment that can drive innovation while respecting privacy and ethical norms.

To effectively address the identified gaps and enhance harmonization with AI governance regulations, it is imperative to:

- Develop global standards for neuroinformatics data sharing that address privacy, ethical use of data, and interoperability. These standards should be robust enough to facilitate data sharing across different domains, particularly in sensitive areas including healthcare.
- Invest in technologies such as differential privacy and federated learning. These investments would enable secure data sharing without compromising individual privacy and help navigate the evolving landscape of data protection regulations.
- Strengthen international collaboration to navigate regulatory disparities and facilitate cross-border data sharing, ensuring that neuroinformatics research can benefit from global data resources and expertise.
- Create specific governance frameworks that address the unique challenges posed by neurotechnological advancements and genetic research, including protections for cognitive privacy and robust consent mechanisms.

## 7 Conclusion

This article systematically examines neuroinformatics within global AI governance, exploring state-of-the-art practices and privacy challenges, assessing AI regulations, and offering strategic recommendations. It emphasizes the crucial need for standardized data sharing and robust ethical frameworks to enhance global research and ensure ethical innovation.

## Footnotes

^1^Due to the 2000-word limit for opinion articles, we cannot present this topic in full depth.
